# Structural Covariance Analysis Reveals Differences Between Dancers and Untrained Controls

**DOI:** 10.3389/fnhum.2018.00373

**Published:** 2018-09-25

**Authors:** Falisha J. Karpati, Chiara Giacosa, Nicholas E. V. Foster, Virginia B. Penhune, Krista L. Hyde

**Affiliations:** ^1^International Laboratory for Brain, Music and Sound Research (BRAMS), Montreal, QC, Canada; ^2^Faculty of Medicine, McGill University, Montreal, QC, Canada; ^3^Department of Psychology, Concordia University, Montreal, QC, Canada; ^4^Department of Psychology, Université de Montréal, Montreal, QC, Canada

**Keywords:** dance, music, gray matter, structural covariance, dorsolateral prefrontal cortex

## Abstract

Dancers and musicians differ in brain structure from untrained individuals. Structural covariance (SC) analysis can provide further insight into training-associated brain plasticity by evaluating interregional relationships in gray matter (GM) structure. The objectives of the present study were to compare SC of cortical thickness (CT) between expert dancers, expert musicians and untrained controls, as well as to examine the relationship between SC and performance on dance- and music-related tasks. A reduced correlation between CT in the left dorsolateral prefrontal cortex (DLPFC) and mean CT across the whole brain was found in the dancers compared to the controls, and a reduced correlation between these two CT measures was associated with higher performance on a dance video game task. This suggests that the left DLPFC is structurally decoupled in dancers and may be more strongly affected by local training-related factors than global factors in this group. This work provides a better understanding of structural brain connectivity and training-induced brain plasticity, as well as their interaction with behavior in dance and music.

## Introduction

Investigating the brains of individuals with specialized training, such as dancers and musicians, provides insight into training-associated brain plasticity as well as brain-behavioral relationships. Dance and music share fundamental similarities, including their reliance on sensorimotor integration as well as the structured and easily quantifiable nature of the training process. However, dance and music training also differ. For example, dance training commonly involves whole-body movements and following sound, while music training generally employs effector-specific movements to produce sound. The comparison of the neural correlates of dance vs. music can further understanding of brain characteristics related to auditory-motor artistic training in general, as well as characteristics that reflect more unique demands of the two types of training. Many studies have investigated the neural correlates of music training (for reviews, see Herholz and Zatorre, 2012; Schlaug, 2015), and there is growing interest to study the neural correlates of dance (e.g., Bläsing et al., 2012; Karpati et al., 2015, 2017; Bar and DeSouza, 2016; Di Nota et al., 2016; Giacosa et al., 2016). The present study aims to build on this literature by applying structural covariance (SC) analysis to investigate interregional gray matter (GM) structural relationships in dancers and musicians relative to untrained controls.

The neural correlates of dance and music have been examined using a wide variety of neuroimaging techniques (e.g., structural MRI, functional MRI, PET, fNIRS) and, within these techniques, a variety of measures and analysis methods (e.g., surface-based morphometry, voxel-based morphometry, diffusion tensor imaging). For example, studies comparing local GM structure between experts and non-experts have found differences in widespread areas including auditory and motor regions (e.g., Amunts et al., 1997; Schlaug, 2001; Schneider et al., 2002; Gaser and Schlaug, 2003; Bermudez et al., 2009; Han et al., 2009; Hänggi et al., 2010; Elmer et al., 2013; Fauvel et al., 2014; James et al., 2014; Nigmatullina et al., 2015; Karpati et al., 2017). Our study directly comparing dancers vs. musicians found that, although both differed from untrained controls, they did not differ from each other in local GM structure (Karpati et al., 2017). GM structure has also been demonstrated to correlate with performance on dance- and music-related tasks (e.g., Foster and Zatorre, 2010b; Karpati et al., 2017).

In contrast, differences between dancers and musicians were found in white matter (WM). Dancers demonstrated reduced fractional anisotropy (FA), suggesting reduced fiber coherence and increased fanning or crossing fibers, in interhemispheric, motor and sensorimotor integration tracts (Giacosa et al., 2016). This is consistent with previous work demonstrating increased FA in musicians relative to nonmusicians (e.g., Han et al., 2009; Halwani et al., 2011; Steele et al., 2013; Rüber et al., 2015), and reduced FA in dancers vs. nondancers (Hänggi et al., 2010). This suggests that the characteristics that differentiate these types of training (e.g., focus on whole-body vs. effector-specific movements) may be associated with different patterns of interregional connections.

Further insight into the interregional relationships of dancers and musicians has been provided by functional neuroimaging studies, which have found enhanced resting functional connectivity in a motor control pathway in dancers (Li et al., 2015), and in auditory, motor and somatosensory areas in musicians compared to their untrained counterparts (Luo et al., 2012; Choi et al., 2015; Klein et al., 2016; Palomar-García et al., 2017). Furthermore, activation in sensorimotor regions has been observed during music- (e.g., Gaab et al., 2003; Meister et al., 2004; Bangert et al., 2006; Bengtsson and Ullén, 2006; Foster and Zatorre, 2010a; Kleber et al., 2010; Lee et al., 2011; Klein and Zatorre, 2015) and dance-related tasks (e.g., Calvo-Merino et al., 2005; Brown et al., 2006; Cross et al., 2006; Pilgramm et al., 2010; Tachibana et al., 2011; Jola et al., 2013; Bar and DeSouza, 2016) which is influenced by short- and long-term training (e.g., Bangert et al., 2006; Cross et al., 2009; Foster and Zatorre, 2010a; Kleber et al., 2010; Pilgramm et al., 2010; Lappe et al., 2011). Taken together, these studies have observed a relationship between both brain structure and interregional connectivity with dance and music. They suggest that structure and interregional relationships may be influenced by training and associated with dance and music-related skills. SC analysis will build on these findings by identifying cortical regions that show unique relationships with overall GM structure, which may indicate regions that are influenced by local training-related factors.

The SC analysis used in the present study is based on the Mapping Anatomical Correlations Across Cerebral Cortex (MACACC) method developed by Lerch et al. (2006). This analysis measures how the GM structure of one brain area correlates with structure of other areas, and such correlations can be compared between groups or correlated with a behavioral measure (Lerch et al., 2006). Interregional correlations as measured by SC analysis show some consistency (35%–40%) with other measures of connectivity, such as WM tractography maps and functional resting state networks. Given this proportion of consistency, SC analysis has been demonstrated to provide unique information regarding interregional relationships (Lerch et al., 2006; Gong et al., 2012; Clos et al., 2014; Hardwick et al., 2015; Reid et al., 2016). Additional factors that may contribute to SC findings include indirect WM connections and indirect functional connectivity (i.e., parallel paths; Evans, 2013; Reid et al., 2016). Of particular importance to the present study, SC findings may also be related to mutual trophic influences on the connected regions (Ferrer et al., 1995; He et al., 2007; Cohen-Cory et al., 2010; Evans, 2013) and such influences may be affected by experience-related brain plasticity (Draganski and May, 2008; Evans, 2013; Lövdén et al., 2013; Kolb and Gibb, 2014). Therefore, SC findings may be related to a combination of WM tracts, synchronous neuronal firing, and mutual trophic influences between the correlated regions.

Many studies have employed SC analysis using a seed-based approach, where correlations were examined between structure (e.g., cortical thickness, CT) in a predetermined seed region and structure in other brain regions. These studies have investigated SC in clinical populations (e.g., Raznahan et al., 2010; Bernhardt et al., 2014; Voss and Zatorre, 2015; Zhao et al., 2015; Sharda et al., 2016, 2017) or general healthy groups (e.g., Camilleri et al., 2015; Hardwick et al., 2015). Only one study has applied SC analysis to a trained population. Bermudez et al. (2009) applied a seed-based MACACC analysis to a sample of musicians and non-musicians. They found that, in the non-musicians, the area of the brain where CT was correlated with CT in right frontal seed regions was more expansive than in musicians. This suggests that trained groups show a different CT covariance profile relative to untrained groups, which may be modulated by factors associated with training-related brain plasticity. It supports the idea that SC analysis provides additional information regarding the brain structural characteristics of trained populations that cannot be examined using only group comparisons of regional structure.

Although the use of a seed-based approach allows the investigation of specific brain networks or *a priori* regions of interest, it is also limited by the choice of specific regions. Another method, designed by Lerch et al. (2006), avoids any limitation or bias arising from seed selection by instead examining the SC of each vertex across the whole brain with each other vertex. This provides a measure of the relative interconnectivity of each vertex. The correlation of structure at each vertex to each other vertex is very computationally expensive, however a proxy measure involving correlating structure at each vertex to the individual’s mean structural measure across the whole brain (e.g., mean CT) has been developed (Lerch et al., 2006) and replicated (Lee et al., 2014a). Although the proxy method is not perfectly identical to the full method as a result of unequal standard deviations of CT at each vertex (Lerch et al., 2006), the two studies listed above directly compared the two methods and found a nearly identical pattern of results, leading to the conclusion that the proxy method is an appropriate substitute for the full method. This seed-free method has been used to investigate the relationship between SC and cognitive task performance in adolescents (Lee et al., 2014a,b), but it has not yet been applied to any trained populations.

In the present study, we apply this method to investigate the relationship of SC with dance and music. The objectives of this study were to: (1) test for differences in SC between expert dancers, expert musicians and untrained controls; and (2) examine the relationship between SC and performance on dance- and music-related tasks. This will provide further understanding of the brain structural characteristics of these groups, as well as training-associated brain plasticity in general, by testing for regions where GM structure may have a unique relationship with overall GM structure. This can indicate regions that may have increased or decreased influence from the same factors as the rest of the cortex. Those regions that are relatively less influenced by such whole-brain factors may be more influenced by local training-related factors.

Based on previous findings of local GM structure, WM and functional connectivity differences among dancers, musicians and untrained controls in sensorimotor regions (e.g., Bermudez et al., 2009; Li et al., 2015; Rüber et al., 2015; Giacosa et al., 2016; Karpati et al., 2017; Palomar-García et al., 2017) group differences in SC were expected in similar sensorimotor regions. A reduced correlation between GM structure in sensorimotor regions and the rest of the cortex was expected in the trained groups as an indication of the influence of local training-associated factors on these regions. Complementary findings were expected in the brain-behavioral analysis, with regions showing SC differences in dancers and musicians also demonstrating a relationship between SC and dance- and music-related tasks, respectively. Since SC provides a unique measure relative to WM or functional connectivity, (e.g., Gong et al., 2012; Alexander-Bloch et al., 2013; Evans, 2013; Reid et al., 2016), additional SC differences were expected in brain areas that have not previously been detected studies comparing such traits between dancers, musicians and controls.

## Materials and Methods

### Participants

Three groups of participants (aged 18–40 years old) were recruited for this study: expert dancers (*N* = 20), expert musicians (*N* = 19) and a control group of non-musicians/non-dancers (*N* = 20; Table 1). Dancers and musicians were either currently practicing as professionals or were students involved in professional training programs. Their training was assessed via a detailed questionnaire developed in our laboratories (Bailey and Penhune, 2010; Coffey et al., 2011). Dancers and musicians had on average approximately 15 years of experience in their respective disciplines, and controls had on average less than 1 year of experience in dance, music, figure skating and aerobics. All participants were physically active (e.g., biking, running, or other fitness activities). Dancers were currently practicing contemporary dance as their principal style, but had a variety of training backgrounds including ballet, tap, jazz, swing and ballroom. Dancers whose main style was too similar to the dance task used here (i.e., urban, street or hip-hop) were excluded. Musicians had various instrumental backgrounds, including keyboard instruments, strings, woodwinds, brass and percussion. None of the musicians had absolute pitch. Since the dance task was based on a video game, participants were screened for experience with dance video games; 56 out of 59 participants reported that they never or rarely (up to three times per year) played dance video games. The remaining three participants (one dancer and two musicians) reported a maximum 4 months of lifetime experience with dance video games. The groups did not differ in age, sex distribution, body mass index (BMI) or level of education (Table 1). Participants had no past or current learning or developmental disorder, neurological or psychiatric condition, or alcohol or substance abuse. This study was carried out in accordance with the recommendations of the Research Ethics Board at the Montreal Neurological Institute and Hospital with written informed consent from all subjects. All subjects gave written informed consent in accordance with the Declaration of Helsinki. The protocol was approved by the Research Ethics Board at the Montreal Neurological Institute and Hospital.

**Table 1:** Participant characteristics.

Group	*N*	Age (years ± SD)	Sex	Body mass index (BMI; ± SD)	Years of dance training (± SD)	Years of music training (± SD)	Level of education (± SD)
Dancers (D)	20	25.1 ± 3.9	14 F, 6 M	21.7 ± 2.2	15.3 ± 5.2	1.8 ± 1.9	2.35 ± 0.6
Musicians (M)	19	22.9 ± 3.4	12 F, 7 M	22.5 ± 3.2	1.0 ± 1.8	15.4 ± 3.4	2.32 ± 1.0
Controls (C)	20	25.4 ± 5.1	13 F, 7 M	21.8 ± 3.2	0.4 ± 0.8	0.5 ± 1.0	2.6 ± 1.1
Comparison between groups		*F*_(2,56)_ = 2.1		*F*_(2,55)_ = 0.38	*F*_(2,56)_ = 135.1	*F*_(2,55)_ = 251.2	*F*_(2,56)_ = 56
		*p* = 0.13		*p* = 0.68	*p* < 0.0001	*p* < 0.0001	*p* = 0.57
		D = M = C		D = M = C	D > M (*p* < 0.0001) )	M > D (*p* < 0.0001)	D = M = C
					D > C (*p* < 0.0001)	M > C (*p* < 0.0001)
					M = C (*p* = 1)	D = C (*p* = 0.27)

*F, females; M, males; SD, standard deviation. Education levels for each participant were calculated on a scale of 1–5, where 1 is the lowest (completed high school) and 5 is the highest (completed doctorate degree). Republished with permission from Karpati et al. (2017)*.

### Behavioral Testing

Participants completed a dance-related task (dance imitation) and a music-related task (melody discrimination). The dance imitation task required participants to imitate seven dance routines of increasing levels of complexity. These routines were selected from the video game Dance Central for Xbox Kinect version 1 (Harmonix^1^). This task assesses the ability to observe and imitate whole body dance movements in real time with music, and was scored using a measure of percent moves correct provided by the Kinect system.

In the melody discrimination task (Foster and Zatorre, 2010a, b), participants were asked to determine if pairs of melodies were the same or different based on changes in pitch. Participants completed four blocks of 30 trials each. This task measures auditory processing and pitch discrimination, and was scored using a measure of percent trials correct. Additional details about these tasks as well as behavioral analyses on this sample have been reported in an earlier study (Karpati et al., 2016).

### MRI Acquisition, Processing and Cortical Thickness Extraction

T1-weighted brain images were acquired for all participants at the Montreal Neurological Institute (MNI) on a 3T Siemens Trio MR scanner with a 32-channel head coil. MRI scanning parameters were as follows: echo time = 2.98 ms, repetition time = 2,300 ms, voxel size 1 mm × 1 mm × 1 mm. Earplugs and headphones were used to reduce noise perception, and foam pads were used to reduce head motion.

Images were processed using the CIVET pipeline (version 1.1.11, Ad-Dab’bagh et al., 2006,^2^). They were registered to the ICBM152 nonlinear model (Collins et al., 1994; Grabner et al., 2006) with 12 degrees of freedom for registration, and corrected for signal intensity nonuniformity (Sled et al., 1998). Images were segmented into GM and WM, cerebrospinal fluid and background (Zijdenbos et al., 1998; Tohka et al., 2004). Deformable models were fitted to the images in order to extract the boundaries between GM and each of WM and cerebrospinal fluid (MacDonald et al., 2000; Kim et al., 2005), resulting in two surfaces with 81,920 polygons each. Following the surface extraction, participants’ cortical mid-surfaces (calculated using the mid-points of the linked inner and outer surfaces) were nonlinearly aligned using the SURFTRACC algorithm and a depth-potential function to a hemisphere-unbiased iterative surface template in order to establish intersubject vertex correspondence (Robbins et al., 2004; Lyttelton et al., 2007; Boucher et al., 2009). Then, a CT map was calculated for each participant, where CT (the distance between the pia mater and GM/WM boundary) was measured at each vertex using the t-link metric (Ad-Dab’bagh et al., 2005; Lerch and Evans, 2005) and then blurred with a 20 mm surface-based blurring kernel (Chung and Taylor, 2004).

### Structural Covariance (SC) Analysis

In order to measure the overall correlation strength of CT of each vertex with all other vertices, a proxy measure of the correlation between vertex CT and whole-brain mean CT was used (Lerch et al., 2006; Lee et al., 2014a,b). This measure of SC was compared between groups (dancers, musicians and controls) at each vertex across the whole brain, using an *F*-test for the presence of a group by mean CT interaction (Eq. 1) followed by pairwise comparisons using the same general linear model.

Following the finding of a significant cluster in the above *F*-test, additional *post hoc* tests were conducted to investigate several factors that may contribute to this result. This included:

1. An ANOVA for group differences in whole-brain mean CT to investigate whether global factors affecting whole-brain CT may differ between groups.
2. A region-of-interest test for group differences in CT in the observed cluster using a general linear model to conduct an *F*-test (Eq. 2). This tested for subtle group differences in local structure in this area, which may indicate training-associated plasticity processes acting in this area that were not identified using the whole-brain analysis of local structure reported previously (Karpati et al., 2017).
3. Levene’s test for group differences in variance of CT in this cluster, since correlations are less likely to occur with a variable that has low variance.
4. An *F*-test for group differences in the correlation between CT in this cluster and CT in all other vertices, as well as a qualitative examination of correlations with cluster CT across the whole brain in each group. This allowed for a comparison of the location and extent of areas where CT is correlated with cluster CT, thereby supporting findings of overall increased or decreased whole-brain correlation with the cluster.

The relationship between SC and performance on the dance imitation and melody discrimination tasks was also investigated. Across all participants, an interaction between task score and mean CT was tested at each vertex across the whole brain (Eq. 3) for each task separately.

All analysis was conducted using SurfStat software^3^. Age, sex and a proxy measure of brain volume (pBV, Karama et al., 2011) were included as covariates. Vertex clusters were defined using a forming threshold of *p* < 0.01, and correction for family-wise error at *p* < 0.05 was then applied at the cluster level using random field theory (Friston et al., 1994). Effect sizes for significant results were measured as partial eta squared ($\eta_{\text{p}}^{2}$; Lakens, 2013).

(1) $${\text{V}}_{\text{ertexCT}}=1+\text{Group + MeanCT + Age + Sex + pBV + Group * MeanCT}$$

(2) Cluster MeanCT = 1 + Group + Age + Sex + pBV

(3) VertexCT = 1 + Score + MeanCT + Age + Sex + pBV + Score * MeanCT

## Results

### Group Comparison

**Figure 1:**
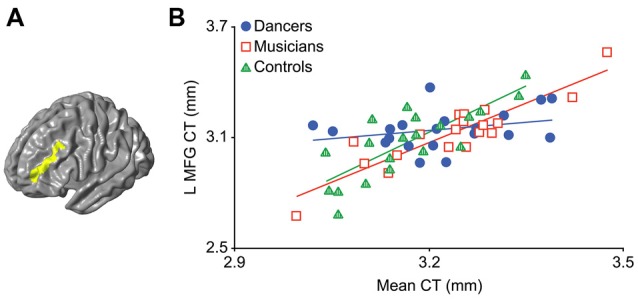
The result of the *F*-test for the presence of a group by mean cortical thickness (CT) interaction is shown in panel **(A)** demonstrating a significant cluster in the left middle frontal gyrus (MFG; *p* = 0.03). In panel **(B)** a scatterplot shows the relationship between mean CT and CT in this cluster (i.e., mean CT of the cluster, adjusted for covariates) in the dancers, musicians and controls. Dancers have a significantly lower correlation between mean CT and left MFG CT compared to controls.

An *F*-test (*df* = 2, 50) for the presence of a group (dancer, musician or control) by mean CT interaction yielded a significant cluster in the left middle frontal gyrus (MFG; *p* = 0.03; Figure 1A; Table 2). This cluster will be referred to as the Group MFG Cluster. Individual group maps of mean CT-regional CT relationship are included as Supplementary Figure S1. Pairwise comparisons (*df* = 50) show that this MFG cluster result is driven by a reduced correlation between mean CT and regional CT in this area in the dancers compared to the controls (*p* < 0.01; Table 2). No significant differences in the strength of the mean CT-regional CT correlation were found between musicians and either dancers or controls. The relationship between mean CT and CT in this cluster was *r* = 0.29 (*p* = 0.2) for dancers, *r* = 0.9 (*p* < 0.0001) for musicians, and *r* = 0.8 (*p* < 0.0001) for controls (Figure 1B). Effect size calculations indicated a partial eta squared of 0.30 for the *F*-test cluster and 0.29 for the pairwise cluster (Table 2), corresponding to a large effect size (Cohen, 1988).

**Table 2:** Group differences in structural covariance (SC).

Analysis	Cluster			Peak (MNI coordinates)						
	*P*-value (2-tailed)	Extent	Effect size ($\eta_{\text{p}}^{2}$)	Brain region	Brodmann area	*x*	*y*	*z*	*F*-value (*df* = 2, 50)	Effect size ($\eta_{\text{p}}^{2}$)
*F*-test	0.03	534 vertices/1,371 mm^2^	0.30	L MFG	46	−35	51	22	12.65	0.34
				L SFS	10	−30	51	11	10.14	0.29
				L MFG	9	−35	33	38	6.40	0.20
									***t*-value (*df* = 50)**	
Dancers < controls pairwise comparison	0.001	1,222 vertices/3,090 mm^2^	0.29	L MFG	46	−35	51	22	4.70	0.31
				L MFG	9	−35	32	38	3.58	0.20
				L SFG	9	−16	51	37	3.44	0.19
				L SFS	9/46	−25	41	25	3.13	0.16
				L MFG	9	−46	25	34	3.01	0.15

*Clusters represent regions where there is a group difference in correlation between regional CT and mean CT. Clusters are significant at *p* < 0.05 after correction for family-wise error using random field theory. CT, Cortical thickness; MFG, Middle frontal gyrus; SFG, Superior frontal gyrus; SFS, Superior frontal sulcus*.

### *Post Hoc* Tests of the Group Comparison

**Figure 2:**
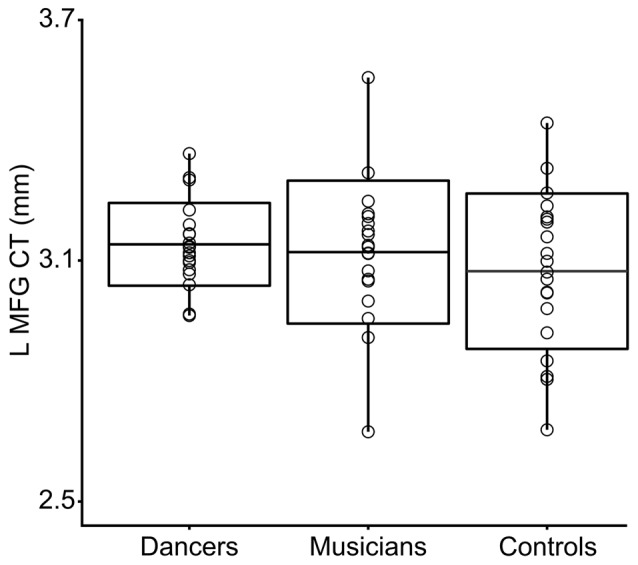
This box plot shows the distribution of CT in the Group MFG Cluster (i.e., mean CT of the cluster, adjusted for covariates) for each group. Boxes represent means and standard deviations. There is a trend towards a group difference in variance in CT in this region (Levene’s test *p* = 0.07). Pairwise comparisons showed that this trend is driven by reduced variance in the dancers compared to controls (Levene’s statistic = 6.9, *p* = 0.01).

**Figure 3:**
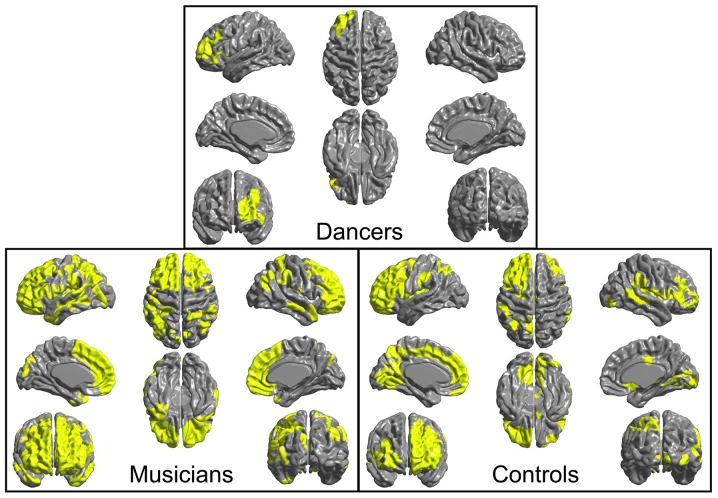
Regions where CT is correlated (cluster *p* ≤ 0.05) with CT in the left MFG cluster (i.e., mean CT of the cluster, adjusted for covariates) are shown in yellow for each group. In both musicians and controls, CT in this cluster is significantly correlated with CT in a variety of bilateral regions. In contrast, in the dancers, CT in this cluster is not correlated with CT in any areas outside of the left frontal lobe.

Following the significant finding above, further testing was conducted to characterize variation in whole brain mean CT and MFG cluster CT separately:

1. An ANOVA for group differences in whole-brain mean CT revealed no significant differences (*p* > 0.1).
2. A region-of-interest *F*-test for group differences in CT in the Group MFG Cluster did not show any significant differences (*p* = 0.4).
3. Levene’s test for group differences in variance in CT in the Group MFG Cluster showed a trend towards a group difference (Levene’s statistic = 2.7, *p* = 0.07). Pairwise comparisons showed that this trend is driven by reduced variance in the dancers compared to controls (Levene’s statistic = 6.9, *p* = 0.01). Musicians did not differ significantly from either dancers or controls in variance in CT in this region (Levene’s statistic ≤1.9, *p* > 0.1). The distribution of CT in this region for each group is shown in Figure 2.
4. An *F*-test conducted across the whole brain for the presence of a group by Group MFG Cluster CT interaction did not show any significant results. However, examination of brain regions in which Group MFG Cluster CT is correlated in each group separately showed that, in the musician and control groups, CT in this cluster is significantly correlated with CT in a variety of bilateral brain regions (cluster *p* ≤ 0.05). In the dancer group, CT in this cluster is not correlated with any regions outside of the left frontal lobe. Correlation maps showing regions where CT is correlated with Group MFG Cluster CT for each group is presented in Figure 3.

### Brain-Behavior Analysis

**Figure 4:**
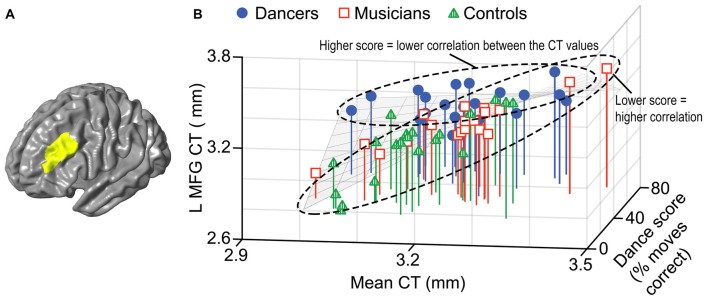
A negative interaction between score on the dance imitation task and mean CT was found in a cluster in the left MFG (*p* = 0.006) as shown in panel **(A)**. In panel **(B)** this interaction is visualized in a 3-dimensional scatterplot showing dance task score, mean CT and left MFG CT (i.e., mean CT of the cluster, adjusted for covariates) in the dancers (blue), musicians (red) and controls (green). This demonstrates that a higher score on the dance task is associated with a reduced correlation between mean CT and left MFG CT.

**Figure 5:**
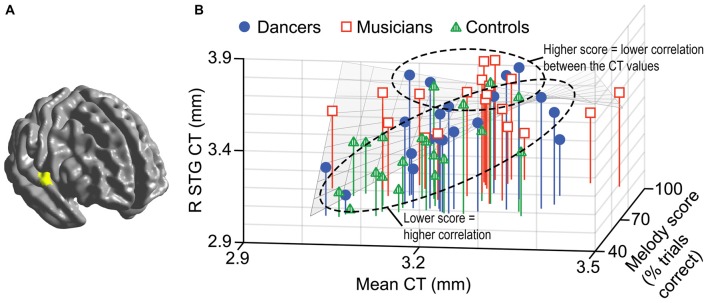
A negative interaction between score on the melody discrimination task and mean CT was found in a cluster in the right superior temporal gyrus (STG; *p* = 0.01) as shown in panel **(A)**. In panel **(B)**, this interaction is visualized in a 3-dimensional scatterplot showing melody task score, mean CT and right STG CT (i.e., mean CT of the cluster, adjusted for covariates) in the dancers (blue), musicians (red) and controls (green). This demonstrates that a higher score on the melody task is associated with a reduced correlation between mean CT and right STG CT.

A general linear model (*df* = 52) testing for an interaction between dance imitation task score and mean CT showed a negative interaction between these two factors in a cluster in the left MFG (*p* < 0.01; Table 3; Figure 4A). This indicates that higher dance task scores are associated with a lower correlation between mean CT and left MFG CT, which is illustrated in Figure 4B. A general linear model (*df* = 52) testing for an interaction between melody discrimination task score and mean CT revealed an interaction between these two factors in a cluster in the right superior temporal gyrus (STG; *p* = 0.01; Table 3; Figure 5A). This indicates that higher melody task scores are associated with a lower correlation between mean CT and right STG CT, which is shown in Figure 5B. Effect size calculations indicated a partial eta squared of 0.23 for the dance cluster and 0.21 for the melody cluster (Table 3), corresponding to a medium effect size (Cohen, 1988).

**Table 3:** SC correlations with behavioral tasks.

Task	Cluster			Peak (MNI coordinates)						
	*P*-value (2-tailed)	Extent	Effect size ($\eta_{\text{p}}^{2}$)	Brain region	Brodmann area	*x*	*y*	*z*	*t*-value (*df* = 52)	Effect size ($\eta_{\text{p}}^{2}$)
Dance imitation	0.006	863 vertices/2,326 mm^2^	0.23	L MFG	46	−37	47	23	4.10	0.24
				L MFG	9	−35	33	38	3.44	0.19
				L SFS	9/46	−26	40	25	3.15	0.16
				L MFG	9	−47	26	32	2.98	0.15
Melody discrimination	0.01	226 vertices/565 mm^2^	0.21	R Heschl’s gyrus	41	49	−11	6	4.19	0.25
				R STG	22	47	−7	−5	3.11	0.16
				R STG	22/42	42	−18	−1	2.59	0.11

*Clusters represent regions where the correlation between regional CT and mean CT is associated with task scores. Clusters are significant at *p* < 0.05 after correction for family-wise error using random field theory. CT, Cortical thickness; MFG, Middle frontal gyrus; SFS, Superior frontal sulcus; STG, Superior temporal gyrus*.

## Discussion

This study is the first to compare SC (i.e., the relative correlation of structure in each region to overall GM structure) between expert dancers, expert musicians and untrained controls. The relationship between SC and performance on measures of dance- and music-related skills (i.e., dance imitation and melody discrimination tasks) was also examined. Dancers showed a negligible correlation between CT in the left MFG and whole-brain mean CT, in contrast to controls and musicians, whose MFG CT was strongly correlated with mean CT. Across all participants, a reduced correlation between left MFG CT and mean CT was associated with higher scores on the dance imitation task, and a reduced correlation between right STG CT and mean CT was related to better performance on the melody discrimination task. These findings show that SC can provide unique insight into training-associated brain plasticity and brain-behavior relationships. SC analysis allows the identification of regions that may show unique relationships with overall structure in a trained vs. untrained group, thereby indicating regions that may be influenced by local training-related factors.

### Structural Covariance and Dance: Structural Decoupling of the Left DLPFC

Dancers showed a difference in SC relative to untrained controls. Specifically, the correlation between left MFG CT and mean CT was reduced in the dancers. In support of this result, *post hoc* tests showed that left MFG CT was correlated with CT in widespread brain areas in the controls, while it was not correlated with CT in any areas outside of the left frontal lobe in dancers. These findings suggest that this region, localized to the dorsolateral prefrontal cortex (DLPFC; Brodmann areas 9 and 46; Petrides, 2000; Fletcher and Henson, 2001; Krawczyk, 2002; Hoshi, 2006), has a unique relationship with overall GM structure in the dancers. This can be interpreted as a structural decoupling of the DLPFC in dancers (i.e., an independence of GM structure in this area relative to overall GM structure). Consistent with this finding, a reduced correlation between left DLPFC CT and mean CT was associated with higher scores on the dance imitation task. This SC-behavior correlation is likely driven by the difference in the SC of this region between dancers and controls, as described above, as well as enhanced behavioral performance in dancers relative to controls on this task (Karpati et al., 2016). The lack of predicted findings in sensorimotor areas furthers the idea that SC provides complementary but not identical information to local GM comparisons. This suggests that the factors influencing SC may be different from those affecting local GM structure.

SC findings may be driven by a variety of factors, including direct WM connections, synchronous neuronal firing, and training-associated mutual trophic influences (Ferrer et al., 1995; Lerch et al., 2006; He et al., 2007; Cohen-Cory et al., 2010; Gong et al., 2012; Evans, 2013; Clos et al., 2014; Hardwick et al., 2015; Reid et al., 2016). The potential contributions of each of these factors to the current results, including support or lack thereof for each, will be discussed.

#### The Potential Contribution of WM to the SC Findings

Our laboratory has investigated WM in this sample. We found reductions in FA along with increased radial diffusivity in widespread left frontal tracts, including the superior longitudinal fasciculus and corona radiata, in the dancers relative to musicians (Giacosa et al., 2016). This is consistent with observations by Hänggi et al. (2010) of reduced FA in the left MFG in dancers relative to non-dancers. The findings from both these studies are in the area of the observed DLPFC cluster. These findings have been interpreted as increased heterogeneity of fiber orientation or increased fanning/crossing fibers in the dancers (Giacosa et al., 2016). These broad connections have likely developed to meet the demands of whole-body dance training. Although these observations indicate differences in interregional connectivity in left frontal regions in dancers, which supports the present findings, it is not clear exactly how such WM findings relate to SC differences. This is because a relationship between SC and FA or other diffusivity measures has not yet been investigated, and negative SC relationships have shown poor location correspondence with WM tracts in previous work (Gong et al., 2012; Reid et al., 2016). It is possible that WM connectivity is a factor underlying the present result, however further studies comparing SC with WM diffusivity measures are needed to clarify the contribution of WM to such SC findings.

#### The Potential Contribution of Functional Synchronicity to the SC Findings

Synchronous neuronal firing between regions has been suggested to play a role in positive SC relationships (Alexander-Bloch et al., 2013; Evans, 2013), therefore a negative SC relationship as observed in the present result may be related to independent firing patterns. In the case of the present results, this hypothesis would imply that the DLPFC activates independently of the rest of the cortex in dancers. This is not supported by the only study that has investigated resting state functional connectivity in dancers vs. non-dancers (Li et al., 2015) as they did not find group differences in this area. Given the research conducted to date, there is no evidence to suggest that functional asynchronicity is a contributor to the current findings. However, further work perhaps using a region of interest approach focused on the DLPFC as well as a combination of resting state and task-based analyses, will increase understanding of the structure-function relationship in this area in dancers.

#### The Potential Contribution of Mutual Trophic Influences to the SC Findings

A third factor that may contribute to SC findings is common trophic influences acting on correlated regions (Ferrer et al., 1995; He et al., 2007; Cohen-Cory et al., 2010; Evans, 2013). Regions which receive mutual influences are hypothesized to demonstrate positive SC relationships, therefore a negative SC relationship as found in the present result would indicate different influences between regions. In the present finding, this would suggest that DLPFC structure is influenced by unique factors relative to the rest of the cortex in dancers. This idea is supported by findings of the *post hoc* tests, which demonstrated that dancers (compared to controls) had reduced variance in CT in this area. This suggests that there may be an optimal range of CT in this region for dance performance, and unique factors may work to ensure this regional CT is achieved regardless of CT across the rest of the brain. These unique factors that may be acting on the DLPFC in dancers are likely due to their training, which is consistent with previous reports of effects of experience- and training-related brain plasticity on brain structural trophic factors (Zatorre et al., 2012; Lövdén et al., 2013; Kolb and Gibb, 2014). Neuroplasticity is associated with a chain of interacting events beginning with changes in gene expression, which in turn may lead to alterations in the expression of growth factors such as brain-derived neurotrophic factor (BDNF; Zatorre et al., 2012; Lövdén et al., 2013; Kolb and Gibb, 2014). Findings of a relationship between BDNF expression and CT (Yang et al., 2012; Legge et al., 2015; Song et al., 2015; Na et al., 2016) support the idea that training-associated brain plasticity may play a role in the structural decoupling of CT in the DLPFC in dancers. Although it is possible that there are several factors contributing to the current result, the idea of local training-related factors acting on this region in the dancers appears to be the most plausible and justified by previous work.

#### The DLPFC and Dance

The DLPFC is known as a region involved in executive processing functions applicable to many domains (e.g., Rowe et al., 2000; Krawczyk, 2002; Petrides, 2005; Champod and Petrides, 2007). Activity in the DLPFC has been observed in many movement-related tasks, including action observation with intent to imitate (Decety et al., 1997; Buccino et al., 2004; Krüger et al., 2014), action planning and selection (Buccino et al., 2004; Hoshi, 2006; Ubaldi et al., 2015), action prediction (Cross et al., 2013) and motor imagery (Gerardin et al., 2000; Grèzes and Decety, 2001; Malouin et al., 2003; Mizuguchi et al., 2013; Sauvage et al., 2013; Wriessnegger et al., 2016). Although these are functions that everyone executes on a daily basis, dancers likely execute them more accurately (e.g., Washburn et al., 2014), frequently and consciously than untrained controls. For example, motor imagery is often integrated into dancers’ training (Overby, 1990) and they spend large amounts of time learning movements through imitation (Harbonnier-Torpin and Barbier, 2012). This may lead to a need for a particular structure of this area in dancers in order to accommodate their unique needs for these types of functions. Additional support for the importance of the DLPFC in dance comes from the finding that internal representations of movements are generated in the DLPFC (Arnsten and Jin, 2014) and have been found to be different in dancers vs. non-dancers (Bläsing et al., 2009). Further work comparing activity in the DLPFC between dancers and untrained controls across a battery of motor, perceptual and cognitive tasks, and how this relates to structure, will help to clarify the role of this region in dance.

### Structural Covariance and Music: Relationship Between the Right STG and Melody Discrimination

In contrast to the difference in SC observed between dancers and controls, the SC of musicians was not found to be significantly different from either dancers or controls. The only previous study to investigate SC in musicians found a difference is SC between musicians and non-musicians using a seed-based approach focused on right frontal regions (Bermudez et al., 2009). The present study employed an unbiased, seed-free whole-brain approach, which may reveal different aspects of SC. The finding of a significant difference in SC between dancers and controls without a difference between musicians and controls is consistent with previous findings from our laboratory of differences in interregional connections in WM between dancers and musicians (Giacosa et al., 2016). Taken together, these findings suggest that musicians may have unique interregional brain structural relationships relative to dancers and controls, and their SC of the DLPFC as measured in the present study are intermediate between those of dancers and controls. Future work comparing functional connectivity in these groups is necessary to further distinguish their interregional relationships.

Although no group differences in SC were found between musicians and either dancers or controls, a relationship between SC in the right STG and performance on a music-related task (i.e., melody discrimination) was found. A reduced correlation between CT in the right STG and mean CT was associated with better scores on the melody discrimination task. This is consistent with previous findings of, and may be driven by, increased CT in the right STG in musicians vs. non-musicians (Bermudez et al., 2009; Karpati et al., 2017), better performance on this melody task in musicians vs. non-musicians (Foster and Zatorre, 2010a; Karpati et al., 2016), and a positive correlation between right STG CT and melody task performance (Karpati et al., 2017).

### Conclusions and Future Directions

This study is the first to employ a seed-free method of SC analysis in trained populations, and to examine the relationship between SC and dance. SC was compared between expert dancers, expert musicians and untrained controls; and correlated with performance on dance- and music-related tasks. The left DLPFC was found to be structurally decoupled in dancers, and SC in this area was related to performance on the dance imitation task. These findings suggest that the left DLPFC may be more influenced by local training-related factors, relative to global factors that affect overall brain structure, in dancers. They also indicate that training-associated brain plasticity may extend beyond regional group differences in brain structure and into the structural relationship between regions.

This work provides a better understanding of the neural correlates of music and dance, training-related structural brain plasticity, and brain-behavior relationships. Future longitudinal studies to differentiate pre-existing brain structural characteristics vs. those caused by training will provide further insight into these concepts. This work can be applied to the development of arts-based therapies for clinical populations. Understanding how dance and music training may influence brain structure, and the regions influenced, would allow for the design of specific therapies in order to target regions and brain processes that would be most beneficial for particular populations.

## Author Contributions

Conception and experimental design of this study was conducted collaboratively by FK, CG, NF, VP and KH. FK and CG recruited participants, and collected the behavioral and neuroimaging data. FK analyzed the data with guidance from NF. FK led the result interpretation, with input from CG, NF, VP and KH. FK wrote the manuscript draft. FK, CG, NF, VP and KH contributed to manuscript revision.

## Conflict of Interest Statement

The authors declare that the research was conducted in the absence of any commercial or financial relationships that could be construed as a potential conflict of interest.

## Footnotes

^1^http://www.harmonixmusic.com

^2^http://www.bic.mni.mcgill.ca/ServicesSoftware/CIVET1112

^3^http://www.math.mcgill.ca/keith/surfstat/
