# Supplementary material for: Structural Covariance Analysis Reveals Differences Between Dancers and Untrained Controls
Source: Front Hum Neurosci. 2018 Sep 25;12:373. doi: 10.3389/fnhum.2018.00373 (PMC6167617; doi:10.3389/fnhum.2018.00373)
Supplement: Supplementary file 1 [file Image_1.PDF]

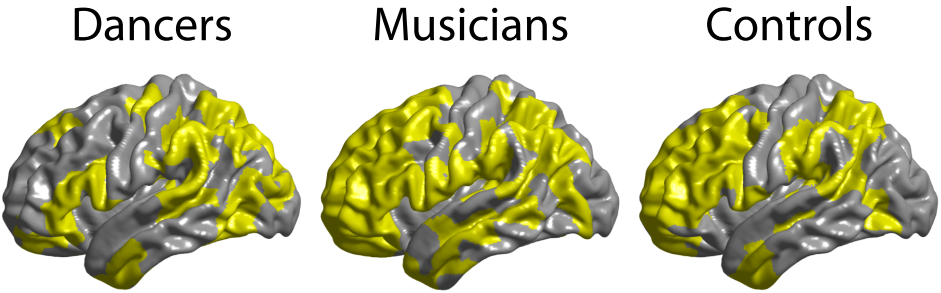

Supplementary Figure 1: Regions having significant correlation between mean cortical thickness (CT) and regional CT, calculated separately by group.
